# Benchmarking OPLS-AA and OpenFF for HDI–PEG Thermoplastic Polyurethanes with Varying Soft-Segment Length

**DOI:** 10.3390/molecules31081259

**Published:** 2026-04-10

**Authors:** Francesco Blasina, Tetiana Bubon, Francesco Cristiano, Giovanna Giuliana Buonocore, Marino Lavorgna, Sabrina Pricl, Mariamelia Stanzione, Domenico Marson, Erik Laurini

**Affiliations:** 1 Molecular Biology and Nanotechnology Laboratory (MolBNL@UniTs), Department of Engineering and Architecture (DIA), University of Trieste, Piazzale Europa 1, 34127 Trieste, Italy; francesco.blasina2@studenti.units.it (F.B.); tetiana.bubon@dia.units.it (T.B.); sabrina.pricl@dia.units.it (S.P.); erik.laurini@dia.units.it (E.L.); 2 Bogolyubov Institute for Theoretical Physics of the National Academy of Sciences of Ukraine, 14-b Metrolohichna Str., 03143 Kyiv, Ukraine; 3 Institute of Polymers, Composites and Biomaterials—National Research Council of Italy, P.le E. Fermi 1, 80055 Portici, Italy; franc.cristiano@studenti.unina.it (F.C.); giovannagiuliana.buonocore@cnr.it (G.G.B.); marino.lavorgna@cnr.it (M.L.); mariamelia.stanzione@cnr.it (M.S.); 4 Department of General Biophysics, Faculty of Biology and Environmental Protection, University of Lodz, ul. Pomorska 141/143, 90-236 Łódź, Poland

**Keywords:** thermoplastic polyurethanes, soft-segment molecular weight, glass transition temperature, elastic modulus, molecular dynamics simulations, OpenFF, OPLS-AA

## Abstract

Thermoplastic polyurethane properties are governed by the interplay between soft-segment mobility, hard-segment interactions, and segmented morphology, yet the extent to which atomistic predictions of their thermal and mechanical behavior depend on force-field choice remains insufficiently benchmarked. Here, we combine FTIR, DSC, TGA, and tensile testing with all-atom molecular dynamics simulations to investigate HDI–PEG polyurethane systems across a controlled soft-segment series. Experimentally, films with PEG molecular weights of 400, 1000, and 1500 g/mol were characterized, while simulations were extended to 400–2000 g/mol to compare two complementary force-field frameworks under a consistent protocol: OPLS-AA, a conventional atom-type-based force field, and OpenFF/Sage, a direct-chemical-perception framework augmented here with bespoke torsional refinements. Both force fields reproduce the composition-driven decrease in Tg and density with increasing PEG length, but differ systematically in absolute values, with OPLS-AA predicting higher densities and Tg values than OpenFF. Tensile experiments show the highest elastic modulus for PEG400, a marked decrease at PEG1000, and a partial recovery at PEG1500. Although nanosecond-scale deformation simulations overestimate absolute moduli because they probe high-rate elastic response, they recover composition-dependent stiffness differences, with OpenFF yielding a more pronounced non-monotonic trend than OPLS-AA. Overall, this work provides an experimentally anchored benchmark for assessing which composition-driven trends in HDI–PEG polyurethanes are robust across force-field families, and which observables remain sensitive to model assumptions and simulation scale.

## 1. Introduction

Polymers are widely used materials, with applications ranging from packaging and biomedical devices to high-performance engineering plastics [1,2]. Among these, thermoplastic polyurethanes (TPUs) constitute a particularly versatile class, exhibiting self-healing behavior and shape-memory properties [3,4,5]. TPUs are block copolymers composed of alternating hard and soft segments [6]. The hard segments typically derive from aliphatic, cycloaliphatic, or aromatic diisocyanates, for example hexamethylene diisocyanate (HDI), isophorone diisocyanate (IPDI), methylene diphenyl diisocyanate (MDI), and toluene diisocyanate (TDI), and are often chain-extended with low-molecular-weight diols or diamines, whereas the soft segments are based on polyols such as polyethers, polyesters, or polycarbonates, commonly with molecular weights up to ~4000 g/mol [7,8,9,10].

The macroscopic properties of these materials stem from their segmented architecture. Thermodynamic incompatibility between the rigid hard segments and the flexible soft segments promotes microphase separation, yielding hard, nanostructured domains dispersed within a soft matrix [3,11,12,13,14]. This morphology can be further tuned by post-synthesis thermal treatments such as annealing, which, when carefully optimized, can enhance microphase organization and improve mechanical performance [15,16,17,18,19]. The hard domains act as physical cross-links and reinforcing elements that largely determine strength, whereas the soft segments impart elasticity and extensibility [3,10,19,20,21]. Consequently, the balance between these phases is critical for tailoring the polymer’s mechanical and thermal response.

Among the parameters that control this balance, the soft-segment molecular weight plays a particularly important role. Low-molecular-weight soft segments typically show limited phase separation and higher glass-transition temperatures (Tg), whereas increasing molecular weight promotes microphase separation, lowers Tg, extends the rubbery plateau, and enhances tensile strength [9,22,23,24]. Longer chains may also promote crystallization, leading to more ordered domains that increase the elastic modulus but reduce elongation at break [25]. In polyethylene glycol (PEG)-containing TPUs, low-molecular-weight PEG can act as an efficient plasticizer that reduces stiffness and brittleness, whereas higher-molecular-weight PEG yields stiffer materials, indicating that shorter PEG chains enhance extensibility at equivalent loadings [26,27].

Beyond molecular weight, soft-segment chemistry strongly influences phase morphology and relaxation dynamics. For example, polycarbonate-based soft segments can promote increased phase mixing and higher soft-phase fractions, whereas polyether- and siloxane-based segments more often form well-separated domains favorable for elasticity [28,29]. Consistent with these trends, combined molecular dynamics simulations and experimental studies have shown that soft-segment chemistry and molecular weight jointly govern microphase separation and mechanical behavior in TPUs, with polyether-based systems exhibiting greater phase separation and flexibility than polyester-based counterparts [30]. This difference is generally attributed to the lower rotational barriers and weaker intermolecular interactions of polyether segments, while the ester functionalities in polyester-based systems increase polarity, strengthen interchain interactions, and reduce segmental mobility. These effects are further modulated by hard-segment chemistry, as aliphatic diisocyanate-based systems exhibit substantially greater sensitivity to soft-segment modification than aromatic counterparts [26]. In line with this picture, coarse-grained molecular dynamics simulations have demonstrated that soft-segment chemistry, molecular weight, and hard-segment content collectively control the equilibrium microphase morphology of TPUs, enabling transitions from mixed to strongly phase-separated structures by tuning intermolecular interactions [31]. Overall, TPU properties are governed by the relative content of soft and hard segments and, critically, by how these components interact. Numerous studies have examined the mechanical performance of TPUs and shown that it depends strongly on segment composition and relative proportion [8,24,32,33,34,35,36,37]. In particular, experimental work indicates that increasing hard-segment content enhances microphase separation and mechanical strength by increasing physical cross-link density and hydrogen bonding within hard domains [20,33,35,36]. Complementing these observations, a recent ab initio molecular dynamics study reported that the hard-segment chemical structure governs the strength and reversibility of interchain hydrogen bonding, which correlates with experimentally observed differences in the thermal and mechanical properties of shape-memory polyurethanes [38]. Specifically, symmetric diisocyanates tend to form more ordered, strongly hydrogen-bonded hard domains than asymmetric analogs, resulting in improved mechanical performance [13,34,38,39,40]. However, these rigid domains can also restrict chain mobility, producing stronger but less extensible materials with reduced recoverability and elongation at break [8,24,36,40,41].

Together, these findings underscore that soft-segment chemistry and molecular weight, in combination with hard-segment composition and structure, critically determine TPU morphology and performance. Elucidating structure–property relationships in TPUs is therefore essential for designing materials with optimized properties. In this context, molecular dynamics simulations provide molecular-level insight into polymer structure and thermomechanical behavior, but their predictive accuracy depends strongly on the choice of force-field parameterization, as demonstrated by comparative studies of Tg predictions [42]. At the same time, systematic experimental–computational benchmarks on chemically consistent TPU series remain limited, particularly for studies designed to distinguish composition-driven effects from force-field-dependent offsets while keeping the hard-segment chemistry fixed. Comprehensive evaluations of force-field parameters, supported by direct validation against experimental data, are thus required for reliable simulation-based assessments of polymer behavior.

To address this need, we compare two complementary all-atom force field families within a common simulation workflow. The first is optimized potentials for liquid simulations-all atom (OPLS-AA) [43,44], an all-atom force field based on predefined atom types, originally developed to reproduce conformational energetics and thermophysical properties of organic molecules and now widely used as a transferable baseline for organic and polymeric materials. The second is the Open Force Field (OpenFF) framework [45], a modern, systematically derived parameterization strategy based on direct chemical perception, with broad coverage of chemically heterogeneous motifs such as segmented polyurethanes. In the present work, this comparison is scientifically relevant because the two frameworks differ not only in parameter values, but also in parameterization philosophy, allowing us to test whether key observables are robust across force-field families or remain sensitive to how conformational energetics and intermolecular packing are described. Although other simulation frameworks (including GAFF [46], CGenFF [47], and COMPASS [48]) could in principle also be applied to HDI–PEG systems, the present work was specifically designed as a controlled benchmark between two complementary all-atom parameterization philosophies, namely OPLS-AA and OpenFF.

By using both force fields across a chemically consistent hexamethylene diisocyanate–PEG (HDI–PEG) series, we establish an experimentally anchored benchmark to assess which composition-driven trends are robust across parameterizations and which observables show systematic force-field-dependent offsets. Accordingly, this study uses a controlled HDI–PEG TPU series, in which the hard-segment chemistry based on hexamethylene diisocyanate (HDI) is kept constant while the PEG soft-segment molecular weight is varied from 400 to 2000 g/mol, to examine how chain length influences structural, thermal, and mechanical observables across experiment and simulation. We combine experimental characterization with molecular dynamics simulations, using measured Tg values and elastic moduli to anchor the computational analysis and to evaluate force-field performance against experimentally observed trends. Because microphase separation and PEG crystallization in TPUs can involve length and time scales beyond routine all-atom MD, the present study focuses on observables that are directly accessible within the current simulations, namely density–temperature behavior, chain conformational descriptors, and high-rate elastic response, while using experiments as a reference to benchmark qualitative trends and force-field-dependent offsets. The following sections describe the experimental procedures, simulation setup, and analysis workflows, and then compare structural, thermal, and mechanical trends obtained from the two force fields with the corresponding experimental data. Overall, this work provides a focused benchmark for interpreting atomistic simulations of segmented HDI–PEG TPUs by separating soft-segment-length effects from systematic force-field-dependent differences in predicted properties.

## 2. Results and Discussion

### 2.1. Structural Properties

Fourier-transform infrared spectroscopy was used to verify the chemical structure of the synthesized HDI–PEG TPUs. The absence of the characteristic isocyanate absorption band, together with the presence of urethane-group absorptions associated with N–H, carbonyl, and amide vibrations, is consistent with successful polymerization and formation of urethane linkages in all samples. Minor shifts in the carbonyl and ether bands with increasing PEG molecular weight suggest differences in hydrogen bonding and segmental organization. Overall, the spectra are consistent with the expected chemical structure and confirm effective incorporation of PEG segments with different chain lengths into the polyurethane matrix. Representative FTIR spectra and a detailed analysis are provided in the supporting information (Figure S1.1).

While FTIR confirms the chemical integrity of the polymer chains, it does not provide direct insight into chain conformation or spatial organization. Understanding how polymer chain length, local stiffness, and global conformation are related is essential for rationalizing the structural response of TPU systems. To address this point, we calculated several structural descriptors from the molecular dynamics (MD) simulations—including the end-to-end distance, radius of gyration (Rg), and persistence length—to elucidate how soft-segment molecular weight and force-field choice influence chain conformation and flexibility.

**Figure 1:**
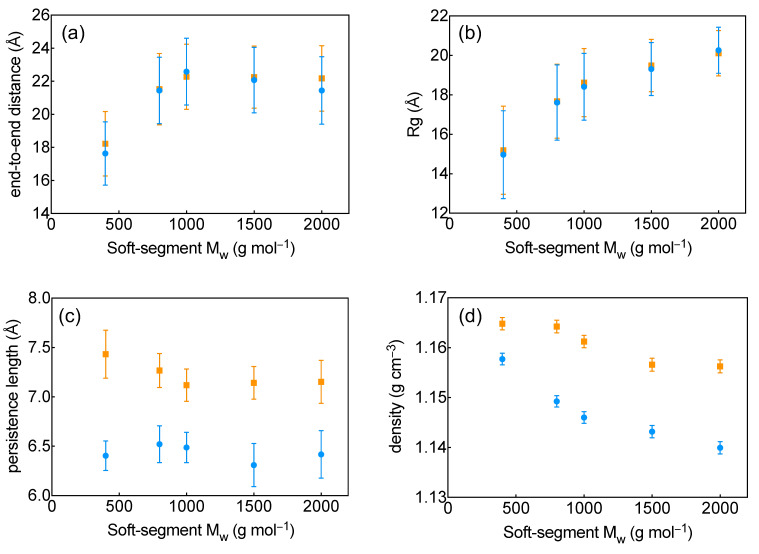
Calculated structural and density-related properties as a function of soft-segment chain length obtained from MD simulations: (**a**) end-to-end distance, (**b**) radius of gyration (Rg), (**c**) persistence length, and (**d**) mass density. Results are shown for the OPLS-AA force field (orange squares) and the OpenFF force field (blue circles), with colored error bars representing statistical uncertainty.

The end-to-end distance results for all modeled HDI–PEG systems are shown in Figure 1a. The analysis indicates that the choice of force field does not significantly affect the calculated end-to-end distances. Instead, chain extension is primarily governed by polymer size rather than force-field parametrization, as supported by statistical testing showing significant differences across chain lengths (one-way ANOVA *p* on the order of 10^−12^), whereas no statistically significant difference was detected between the OpenFF and OPLS-AA force fields (two-sample *t*-test *p* = 0.58). Normality was verified using the Shapiro–Wilk test prior to applying parametric tests. As shown in Figure 1a, PEG-H400 exhibits a noticeably shorter end-to-end distance of approximately 18 Å, whereas the longer chains cluster around ~22 Å. This behavior suggests a marked change between PEG400 and ≥PEG800 in the sampled coil dimensions under the present packing conditions with additional repeat units above the PEG800 contributing only marginally to the overall chain span.

Similarly, the calculated Rg values show no significant dependence on the force field, with both OpenFF and OPLS-AA yielding comparable global chain dimensions (Figure 1b). Accordingly, PEG-H400 differs from the longer chains, whereas intermediate and longer chains exhibit smaller differences and, in some cases, overlapping values. The calculated Rg values range from approximately 15 Å for PEG-H400 to about 20 Å for PEG-H2000. As expected for polymer coils, Rg increases with chain length; beyond ~800 g/mol, this increase becomes more gradual, consistent with polymer scaling behavior.

In contrast, persistence length shows a strong dependence on force-field choice (Figure 1c). Because the persistence-length data did not satisfy normality (Shapiro–Wilk *p* = 0.002), differences between force fields were assessed using a Wilcoxon rank-sum test, which showed a highly significant effect of force field (*p*-value ≈ 10^−17^), whereas no significant differences were detected across chain lengths, suggesting that the persistence length is largely independent of chain length over the range considered. Taken together, these results indicate that force-field choice primarily modulates local stiffness, whereas chain length has a limited effect on local flexibility.

Overall, the analysis highlights a clear separation between global and local effects. Chain length is the primary determinant of global conformation, governing the end-to-end distance and radius of gyration, with a marked change from 400 to 800 g/mol followed by a plateau in end-to-end distance and more gradual growth in Rg beyond ~800 g/mol. By contrast, force-field choice primarily affects local stiffness, as reflected by the persistence length, while producing minimal changes in global dimensions. These findings point to a structural transition in the 400–800 g/mol range, where the polymer evolves from a more compact chain to a mature flexible coil. Despite differences in local stiffness, both force fields predict similar global dimensions, suggesting that parameter differences largely cancel at the level of overall chain conformation.

### 2.2. Density

The mass densities of the HDI–PEG polymer systems obtained from the MD simulations are shown in Figure 1d. For both force-fields, density decreases systematically with increasing soft-segment molecular weight. Using OpenFF, the density drops from approximately 1.155 g/cm^3^ for PEG-H400 to about 1.140 g/cm^3^ for PEG-H2000. A similar trend is reproduced by OPLS-AA, with densities decreasing from roughly 1.165 g/cm^3^ for PEG-H400 to 1.155 g/cm^3^ for PEG-H2000.

Statistical analysis confirms that chain length has a significant effect on polymer density, with pronounced differences between the shorter and longer chains. This dependence appears independent of the force-field, indicating that both parameterizations capture the same qualitative molecular-weight trend. By contrast, the absolute density values show a systematic force-field dependence: across all chain lengths, OPLS-AA predicts consistently higher densities than OpenFF by approximately 0.01 g/cm^3^. This offset is statistically significant, and the applied analysis attributes a measurable contribution of force-field choice to the predicted absolute density.

### 2.3. Glass-Transition Temperature

To analyze the dependence of the Tg on the molecular weight of the PEG soft segment, we employed a combined approach in which experimental differential scanning calorimetry (DSC) measurements provide the reference for assessing force-field performance in molecular dynamics (MD) simulations. Prior to the analysis of thermal transitions, thermogravimetric analysis was performed. The measurements confirmed that all studied TPU systems are thermally stable well above their respective glass-transition and melting temperatures (see Supplementary Materials, Section S1.2 and Figure S1.2), ensuring that the DSC results discussed below are not affected by thermal degradation.

The DSC results (Table 1) show that the PEG-H400 TPU exhibits a glass transition at 271.9 K without a significant endothermic melting peak, indicating that the short PEG soft segments are insufficiently long to promote ordered domains and thus yield a predominantly amorphous structure (see Figure S1.3, left panel). In contrast, PEG-H1000 shows a lower Tg at 230.5 K, followed by a distinct melting endotherm at approximately 299 K, attributable to the development of a semi-crystalline soft phase (Figure S1.3, central panel). Similarly, PEG-H1500 exhibits an even lower Tg at 225.6 K, together with a sharper, more intense melting peak at approximately 307.2 K, consistent with further increases in segmental mobility and crystallization within the soft domains (Figure S1.3, right panel). Overall, the DSC results indicate that increasing PEG molecular weight progressively lowers Tg, reflecting the higher flexibility of longer PEG chains and the associated increase in segmental mobility in the TPU soft phase. At the same time, longer PEG chains promote more pronounced melting endotherms in the PEG-H1000 and PEG-H1500 systems. As reported in early studies [25,49], increasing PEG molecular weight leads to higher melting and crystallization temperatures and more pronounced thermal transitions, consistent with increased crystallinity in TPU films. This trend arises because longer polyol chains more readily form crystalline structures. Despite the presence of urethane bonds between PEG and HDI, the relatively low density of these linkages does not suppress the formation of partially crystalline PEG domains in the present TPU systems.

**Figure 2:**
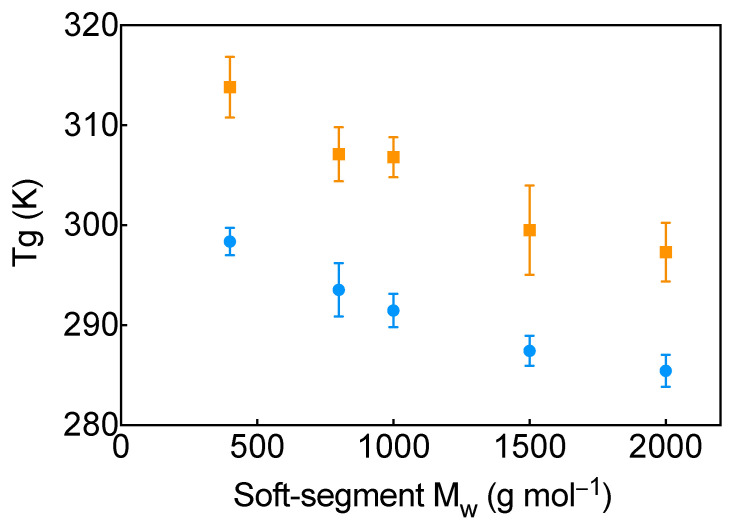
Glass-transition temperature (Tg) as a function of soft-segment chain length obtained from MD simulations. Results are shown for the OPLS-AA force field (orange squares) and the OpenFF force field (blue circles), with colored error bars representing statistical uncertainty.

Figure 2 shows Tg as a function of polymer chain length for all studied systems as obtained from MD simulations. Tg decreases with increasing soft-segment molecular weight, consistent with enhanced segmental mobility in longer chains (see Table 1). Using OpenFF, Tg decreases from approximately 298 K for PEG-H400 to about 285 K for PEG-H2000. OPLS-AA predicts consistently higher absolute Tg values, decreasing from approximately 313 K for PEG-H400 to 297 K for PEG-H2000. This downward trend is strongly supported by the statistical analysis: multiple pairwise comparisons between chain lengths are significant, and the weighted linear model yields a negative coefficient for chain length, confirming a systematic reduction in Tg with increasing polymer size.

In parallel, the choice of force field exerts a pronounced and statistically significant influence on the predicted Tgs. Across all chain lengths, OPLS-AA yields higher Tg values than OpenFF. No significant interaction between force field and chain length is detected, indicating that both force fields capture the same qualitative Tg–molecular-weight dependence despite differences in the absolute temperature scale. Taken together, these results show that chain length and force-field parametrization affect Tg in distinct but complementary ways: chain length governs the decreasing trend, whereas the force field introduces a systematic shift in absolute Tg. The consistent reduction in Tg with increasing polymer length across both force fields suggests that this behavior reflects an intrinsic physical effect rather than a modeling artifact. Overall, these computational results qualitatively agree with the experimental measurements performed on the synthesized TPU films.

Although the Tgs obtained from MD simulations are higher than the experimental values—an expected outcome given inherent limitations of atomistic simulations, including time-scale constraints and the high effective cooling rates—the qualitative agreement in the trends indicates that the simulations capture the key molecular mechanisms governing the thermal behavior of the TPU systems studied here.

### 2.4. Elastic Modulus

The influence of PEG soft-segment molecular weight on the elastic modulus of the TPU systems was assessed using experimental measurements, which serve as the benchmark for evaluating MD simulations and force-field performance. Dynamic mechanical analysis (DMA) was performed on the synthesized TPU films to quantify stiffness (Table 2). Some representative resulting stress–strain curves for the three studied systems are shown in the Supplementary Materials (Figure S1.4), clearly highlighting an initial linear elastic regime. The elastic modulus was determined from the slope of this linear region. The experimental results reveal pronounced differences as a function of PEG soft-segment molecular weight: PEG-H400 exhibits the highest modulus (17.13 ± 3.72 MPa), indicating a stiffer response associated with shorter, less mobile soft segments. In contrast, PEG-H1000 shows a substantially lower modulus (3.73 ± 0.73 MPa), consistent with increased segmental mobility in longer PEG chains. PEG-H1500 displays intermediate behavior (8.05 ± 2.38 MPa), which may reflect the competing effects of enhanced chain flexibility and partial reinforcement arising from soft-segment crystallinity and/or intermolecular interactions.

**Figure 3:**
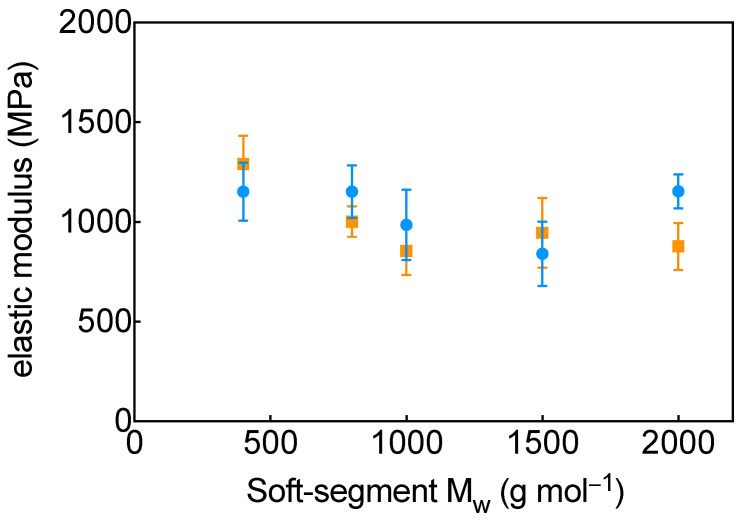
Elastic modulus (E) as a function of soft-segment chain length obtained from MD simulations. Results are shown for the OPLS-AA force field (orange squares) and the OpenFF force field (blue circles), with colored error bars representing statistical uncertainty.

MD simulations were then used to probe how soft-segment molecular weight and force-field choice affect the predicted elastic modulus (Figure 3). The simulated stress–strain curves are provided in the Supplementary Materials (Figures S2.3 and S2.4), where each panel reports the ensemble of bootstrap-resampled curves and the corresponding averaged response. Figure S2.5 reports the bootstrap-derived distributions of elastic modulus values for the investigated systems. For both force fields, all HDI–PEG systems exhibit a clear initial linear elastic region up to ~5% strain, followed by a gradual reduction in slope and plateau-like behavior at higher strains. The moduli obtained from MD are in the range of ~800–2000 MPa (Table 2).

For simulations using OPLS-AA, the elastic modulus generally decreases with increasing PEG molecular weight (Figure 3). PEG-H400 exhibits the highest modulus (~1290 MPa), whereas the lowest value is obtained for PEG-H1000 (~850 MPa). A partial increase is observed for PEG-H1500 (~950 MPa), followed by a decrease for PEG-H2000 (~870 MPa). Overall, the trend can be summarized as: PEG-H400 > PEG-H800 > PEG-H1500 > PEG-H2000 ≈ PEG-H1000.

In contrast, OpenFF predicts a qualitatively different, non-monotonic dependence (Figure 3). The highest moduli are obtained for PEG-H400, PEG-H800, and PEG-H2000 (all ~1150 MPa), whereas the lowest modulus is found for PEG-H1500 (~840 MPa). The corresponding trend is: PEG-H400 ≈ PEG-H800 ≈ PEG-H2000 > PEG-H1000 > PEG-H1500. In this case, lower-molecular-weight soft segments yield higher stiffness, which decreases markedly for PEG molecular weights in the 1000–1500 g/mol range before partially recovering at 2000 g/mol.

Although the elastic moduli obtained from MD are substantially higher than the experimental values, this discrepancy is expected and primarily reflects fundamental differences in time and length scales between atomistic simulations and quasi-static mechanical tests. In MD, deformation is typically imposed at effective strain rates that are many orders of magnitude higher than those accessible experimentally, and the stress response is probed over nanosecond time windows. Under these conditions, polymer chains have limited time to undergo segmental relaxation, disentanglement, and morphological reorganization, which leads to an artificially stiff response and inflated moduli relative to laboratory measurements. In contrast, the experimental force-ramp protocol allows for viscoelastic relaxation during loading, resulting in lower apparent stiffness. Consequently, absolute moduli from MD should be interpreted as high-rate (glassy/non-equilibrated) elastic responses, whereas the experiments probe near-equilibrium behavior at much lower rates. Despite this inherent offset, the comparison remains meaningful for benchmarking force-field performance and assessing relative trends across compositions, because both simulation protocols are applied consistently across the studied systems and capture how changes in soft-segment molecular weight modulate the mechanical response. In light of these considerations, although both force fields predict elastic moduli that are one to two orders of magnitude larger than the experimental values (3–17 MPa), the simulations reproduce key qualitative structure–property relationships. Experimentally, the stiffest TPU corresponds to the shortest soft segment (PEG-H400), the softest response occurs at intermediate PEG lengths (1000–1500 g/mol), and the longest PEG (2000 g/mol) does not yield the minimum modulus. Both force fields capture the high stiffness of PEG-H400 and the absence of a monotonic decrease across the full molecular-weight range. However, the experimentally observed non-monotonic dependence is reproduced with better qualitative agreement by OpenFF, whereas OPLS-AA tends to predict a more monotonic softening. This suggests that the OpenFF parametrization provides a more balanced description of the competing effects of chain flexibility and soft-segment ordering that govern the mechanical response of HDI–PEG TPUs.

## 3. Materials and Methods

### 3.1. Experimental Setup

#### 3.1.1. Chemical Reactants

HDI (98% purity) was purchased from Sigma Aldrich (Darmstadt, Germany) (Chemical Abstracts Service, CAS: 822-06-0), PEG (CAS: 25322-68-3) of different average molecular weights (Mw) 400 (PEG 400), 1000 (PEG 1000) and 1500 (PEG 1500) have been purchased from Sigma Aldrich (Hoeilaart, Belgium), Aldrich Chemical Company Ltd. (Gillingham (Dorset), UK) and Aldrich-Chemie (Taufkirchen, Germany) respectively. The solvent 1,4-Dioxane anhydrous (99.8% purity) (CAS: 123-91-1) was supplied by Sigma Aldrich (Darmstadt, Germany). HDI and all the PEGs were dried under vacuum at 70 °C for 2 h prior use, while 1,4-Dioxane anhydrous was used as received.

#### 3.1.2. Synthesis of HDI–PEG TPUs

The reaction was carried out under an inert and continuous nitrogen atmosphere using a Schlenk line. All formulations were synthesized using a fixed NCO:OH molar ratio of 2:1. A three-necked round-bottom glass flask equipped with a magnetic stirrer was previously conditioned using vacuum/nitrogen cycles and, after 30 min, charged with anhydrous 1,4-dioxane and PEG, the solvent ensured proper control of the reaction mixture viscosity.

The flask was immersed in a thermostated oil bath and maintained at 70 °C under constant stirring at 250 rpm. After thermal equilibration (2 h), HDI was added dropwise to the reaction mixture. The polymerization proceeded at 70 °C under nitrogen for 4 h. Reaction progress was monitored by FTIR spectroscopy through the disappearance of the characteristic absorption band of the isocyanate group (–N=C=O) at approximately 2250 cm^−1^, assigned to the cumulative double-bond stretching vibration. At the end of the reaction, the mixture was poured into a Teflon mold for casting. Solvent removal and subsequent curing were achieved at 80 °C under vacuum overnight affording the final polymeric films. Details of the formulations used for the synthesis of the HDI–PEG polymer films are provided in Table S1.1.

#### 3.1.3. Preparation of HDI–PEG Films

Polymer films with thickness suitable for thermo-mechanical characterization (200–300 μm) were prepared by hot pressing using a Collin P 200 E press (COLLIN Lab & Pilot Solutions GmbH, Maitenbeth, Germany). Approximately 3 g of polymer were placed between two Teflon sheets. The material was first allowed to soften at 200 °C for 4–5 min and subsequently pressed at 100 bar for 5 min at the same temperature. The resulting films were then rapidly cooled by transferring the press plate into water-cooled cassettes, yielding uniform polymer films with homogeneous thickness. A representative photograph of the obtained HDI–PEG film is provided in the Supplementary Information (Figure S1.5).

#### 3.1.4. Chemical Identification by FTIR Spectroscopy

Fourier transform infrared (FTIR) spectra were acquired using a PerkinElmer Frontier FTIR spectrometer (Frontier Dual Range model, PerkinElmer, Waltham, MA, USA) equipped with an attenuated total reflectance (ATR) accessory featuring a diamond/ZnSe crystal and a deuterated triglycine sulfate (DTGS) detector. Spectra were collected at room temperature over the 650–4000 cm^−1^ range, at 4 cm^−1^ resolution, averaging 32 scans per spectrum.

#### 3.1.5. Thermogravimetric Analysis of HDI–PEG Films

The thermal stability and decomposition behavior of the film samples were evaluated by a Q5000 thermogravimetric analyzer (TA Instruments, New Castle, DE, USA), equipped with an automatic sampler. Approximately 7–10 mg of each sample was placed in a platinum pan and heated up to 800 °C at a rate of 10 °C/min under nitrogen atmosphere. The obtained data were analyzed through TA Universal Analysis 2000 software (v. 4.5A, Build 4.5.0.5).

#### 3.1.6. Thermal Properties of HDI–PEG Films

The thermal properties of HDI–PEG film samples, including glass transition temperatures (Tg) and melting temperature (Tm), were analyzed by a DSC Discovery calorimeter (TA Instrument, New Castle, DE, USA) equipped with a quench cooling accessory and an autosampler. Samples (5–7 mg) were sealed in hermetic aluminum pans, with an empty pan used as a reference. Measurements were performed in a heat–cool–heat cycle to eliminate the thermal history of films, over a temperature range from −70 to 210 °C at a heating rate of 10 °C/min. Tg values were determined using TA Universal Analysis software.

#### 3.1.7. Mechanical Properties of HDI–PEG Films

The mechanical properties of the films were evaluated in tensile mode by DMA 850 (Dynamic Mechanical Analyzer) (TA Instrument, Milano, Italy) equipped with an external cooling source of liquid nitrogen. Specimens were prepared with a width of 5 mm and a grip distance of 6 mm, and tests were carried out in force ramp mode at a constant rate of 0.5 N/min at room temperature. Five replicates were tested for each sample, and the results were averaged. Elastic modulus (E) was determined as the slope of the initial linear elastic region of the stress–strain curves. The stress was computed from the measured cross-sectional area, which ranged from 200 to 300 μm thickness.

### 3.2. Molecular Dynamics Simulations

#### 3.2.1. Systems Preparation

**Figure 4:**
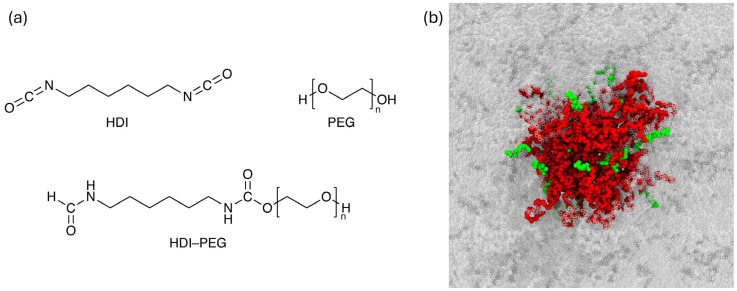
(**a**) Schematic representation of the molecular building blocks of the HDI–PEG polyurethanes investigated in this study. (**b**) Representative initial simulation box for PEG-H2000. Atoms are shown as spheres and colored by chemical identity; hydrogen atoms are omitted for clarity. Semi-transparent replicas indicate periodic images. HDI segments are shown in green (dark gray in replicas) and PEG segments in red (light gray in replicas).

Experimentally, HDI–PEG TPUs were synthesized and characterized for PEG soft-segment molecular weights of 400, 1000, and 1500 g/mol, while atomistic MD simulations were performed for 400, 800, 1000, 1500, and 2000 g/mol to extend the trend analysis beyond the experimentally available formulations (Figure 4a). In the simulated systems, the PEG soft-segment molecular weight was controlled by varying the number of PEG repeat units in each polymer chain, while keeping the HDI-based hard-segment chemistry unchanged. The number of chains in each simulation box was then adjusted as a function of chain length to target an initial density of 1.15 g/cm^3^, yielding box lengths of approximately 4.8 nm. Polymer structures were built in Materials Studio (v. 5.5, BIOVIA, San Diego, CA, USA) software: built chains were packed into cubic boxes using the Amorphous Cell module. Full system compositions are reported in Table S2.1 in the supporting information; a representative structure is shown in Figure 4b.

#### 3.2.2. Force Fields

Two force fields were considered in this study. The first was OPLS-AA [44], for which polymer topologies were generated using the Polymer Parameter Generator (PolyParGen, v. 2) [50]. The second was the Open Force Field (OpenFF) framework (v. 2024.09.0), using the Sage 2.2.1 parameter set [45].

To support the OpenFF-based workflow, a custom Python (v. 3.13) program was developed to automatically generate GROMACS-compatible input files starting from the initial coordinates in Protein Data Bank (PDB) format. The script performs molecular perception, parameter assignment through OpenFF, and system preparation, thereby ensuring a fully reproducible pipeline. The code is openly available in a public GitHub repository (https://github.com/).

For the OpenFF simulations, the force field was further refined using the BespokeFit tool (v. 0.4.0) [51] to refit dihedral parameters against quantum-mechanical reference data for HDI–PEG. No restriction was imposed on the torsions included in the refitting procedure. The reference data were generated with Psi4 (v. 1.9.1) [52], an open-source quantum chemistry software package, using a representative HDI–PEG fragment (SMILES: OC(=O)NCCCCCCNC(=O)OCCOCCOCCOCCOCCOCCOCCOCCO), corresponding to one HDI unit bound to eight PEG repeat units. Quantum-chemical calculations were carried out at the B3LYP-D3BJ/DZVP level, that is, using the Becke three-parameter Lee–Yang–Parr (B3LYP) density functional with Grimme’s D3 dispersion correction and Becke–Johnson damping (D3BJ), together with the DZVP basis set [49,50]. The resulting torsional energy profiles are reported in Figure S2.1.

#### 3.2.3. Simulation Protocol

Molecular dynamics (MD) simulations were performed with the GROMACS simulation package (v. 2025.3) [53]. After steepest-descent minimization, systems were equilibrated in the isothermal–isobaric ensemble for 1 ns at 300 K and 1 bar. Production comprised: (i) heating from 300 to 750 K over 10 ns at 1 bar; (ii) cooling from 750 to 50 K over 140 ns to obtain density–temperature data for Tg estimation; (iii) extraction of the 320 K structure followed by 20 ns equilibration to stabilize the density; and (iv) uniaxial deformation along the z-axis starting from the equilibrated 320 K structure. Deformation was run for 160 ns under semi-isotropic pressure coupling (x, y at 1 bar) with a strain rate of 10^6^ s^−1^, reaching ~15% total strain. A 1 fs timestep was used during heating and cooling, and 2 fs during density and deformation stages. All bonds involving H atoms were constrained with LINear Constraint Solver (LINCS) algorithm [54]. Electrostatics were treated with Particle Mesh Ewald (PME) algorithm [55]; nonbonded and short-range electrostatic cutoffs were 10 Å. Temperature was controlled with the V-rescale thermostat (coupling time constants 1 ps) and pressure with the C-rescale barostat (coupling time constants 5 ps) [56,57]. Periodic boundary conditions were applied in all directions.

Each production workflow (from heating onward) was repeated in 10 independent replicates (with independent initial velocities seeds), and all reported MD observables are presented as the mean across replicas with replica-to-replica uncertainty.

#### 3.2.4. Simulation Analysis

Trajectories were analyzed using GROMACS (v. 2025.3) tools and MDAnalysis (v. 2.9) [58,59] with in-house Python scripts, to compute chain metrics (end-to-end distance, radius of gyration, persistence length), density, glass transition temperature from density–temperature data, and elastic modulus from deformation stress–strain curves.

Thermal noise in the density–temperature curves, particularly pronounced at high temperatures due to large configurational fluctuations, was reduced using principal component analysis (PCA) [60]. Density data were centered and de-composed into orthogonal principal components, retaining the minimum number of components required to preserve the physical trend. All the PCA-processed density–temperature curves are reported in Figure S2.2.

Glass transition temperature was estimated from density–temperature data using global hyperbolic regression following Patrone et al. [61], fitting the full curve to a smooth hyperbola interpolating between glassy and rubbery linear regimes. Tg was taken from the fitted hyperbola (as the temperature corresponding to the hyperbola center). This global fitting procedure improves robustness and reproducibility compared to traditional bilinear extrapolation methods.

Elastic modulus was computed from uniaxial stress–strain curves using an automated workflow [62]. Stress was sampled every 5 ps and denoised via frequency-domain low-pass filtering. The elastic regime was identified using random sample consensus (RANSAC) regression [63], in the region between 0 and 5% strain. Elastic moduli uncertainty was estimated via bootstrap resampling of the 10 independent replicas (1000 bootstrap samples, resampling replicas with replacement). Reported moduli correspond to the mean across bootstrap fits, with uncertainty from the bootstrap distribution.

The effects of soft-segment molecular weight and force-field choice on the investigated descriptors were assessed using a structured statistical analysis framework. Prior to hypothesis testing, data distributions were evaluated for normality using the Shapiro–Wilk test and for homogeneity of variances using F-tests or Levene’s test, as appropriate. For normally distributed data, differences between two groups were assessed using Student’s *t*-tests, while multiple-group comparisons were performed via one-way analysis of variance (ANOVA), followed by Tukey’s honest significant difference (HSD) post hoc tests when significant effects were detected. In cases where normality assumptions were violated, non-parametric Wilcoxon rank-sum tests were employed. When heteroscedasticity was observed or when uncertainties associated with the measured quantities were available, weighted linear models were applied, using inverse-variance weighting to ensure robust parameter estimation.

## 4. Conclusions

We investigated a chemically consistent series of HDI–PEG thermoplastic polyurethanes with varying PEG soft-segment molecular weight using complementary experiments (FTIR, TGA, DSC, and tensile testing) and atomistic MD simulations, with the specific aim of benchmarking two distinct all-atom force-field families, an OPLS-AA-based model and an OpenFF/Sage-based model including bespoke torsion refits, against the same experimental reference. Experimentally, increasing PEG molecular weight decreases Tg and, for PEG1000–PEG1500, produces melting endotherms consistent with crystallizable soft domains in the films. Mechanically, the elastic modulus is highest for PEG400, decreases at PEG1000, and partially recovers at PEG1500, indicating that soft-segment length modulates stiffness in a non-monotonic manner, through competing effects of segmental mobility and structural reinforcement.

In MD, both force fields reproduce the main composition-driven trends in density and Tg, namely a decrease with increasing PEG molecular weight, while showing systematic offsets in absolute values, with the OPLS-AA-based model consistently predicting higher densities and higher Tg values than OpenFF across the series. This indicates that the qualitative dependence of these observables on soft-segment length is robust with respect to force-field family, whereas the absolute thermal scale remains sensitive to parameterization. Chain conformational descriptors further distinguish PEG400 from the longer PEG systems, although these metrics are also influenced by finite-size and packing constraints at the present simulation scale. Uniaxial deformation simulations yield elastic moduli in the 0.8–2.0 GPa range, reflecting the inherently high strain rates and limited relaxation accessible in nanosecond trajectories; these values should therefore be interpreted as high-rate elastic responses rather than quantitative predictions of quasi-static experimental moduli. Within this consistent simulation protocol, however, the force-field comparison remains informative: OpenFF displays a more pronounced non-monotonic dependence of stiffness on PEG molecular weight than the OPLS-AA-based model, in better qualitative agreement with the experimental trend. This difference is relevant not simply because the two models use different parameter values, but because they represent different parameterization philosophies, with OPLS-AA providing an atom-type-based baseline and OpenFF relying on direct chemical perception combined here with bespoke torsional refinement. The observed differences therefore identify which aspects of HDI–PEG TPU behavior are robust across force-field frameworks and which remain sensitive to how conformational energetics and intermolecular packing are described.

Overall, this work provides an experimentally anchored benchmark for atomistic simulations of segmented HDI–PEG TPUs. Rather than claiming fully predictive morphology resolution, it clarifies which observables can already be captured consistently within routine all-atom MD, and which require additional methodological development. In this sense, the present study provides a useful reference both for interpreting simulation results in segmented polyurethane systems and for guiding future force-field refinement and morphology-resolved simulation strategies at longer length and time scales.

## Figures and Tables

**Table 1:** Glass-transition temperature (Tg) values obtained experimentally and calculated from MD simulations using the OpenFF and OPLS-AA force fields. Absolute values and corresponding standard deviations are reported.

System Name	Tg_OpenFF_ (K)	Tg_OPLS-AA_ (K)	Tg_experimental_ (K)
PEG-H400	298.4 ± 1.4	313.8 ± 3.0	271.9 ± 0.22
PEG-H800	293.5 ± 2.7	307.1 ± 2.7	-
PEG-H1000	291.5 ± 1.7	306.8 ± 2.0	230.5 ± 1.50
PEG-H1500	287.5 ± 1.5	299.5 ± 4.5	225.6 ± 1.79
PEG-H2000	285.4 ± 1.6	297.3 ± 3.0	-

**Table 2:** Elastic modulus (E) values obtained experimentally and calculated from MD simulations using the OpenFF and OPLS-AA force fields. Absolute values and corresponding standard deviations are reported.

System Name	E_OpenFF_ (MPa)	E_OPLS-AA_ (MPa)	E_experimental_ (MPa)
PEG-H400	1152.9 ± 145.2	1291.3 ± 142.6	17.13 ± 3.72
PEG-H800	1152.1 ± 131.5	1002.0 ± 77.6	-
PEG-H1000	985.6 ± 177.4	853.8 ± 119.6	3.73 ± 0.73
PEG-H1500	841.6 ± 161.8	945.9 ± 175.7	8.05 ± 2.38
PEG-H2000	1154.5 ± 85.1	877.7 ± 119.0	-

## Data Availability

The original contributions presented in this study are included in the article/Supplementary Materials. Further inquiries can be directed to the corresponding author.

## Supplementary Materials

The following supporting information can be downloaded at: https://www.mdpi.com/article/10.3390/molecules31081259/s1. Experimental details, as well as figures related to the experiments and molecular dynamics simulations discussed in the manuscript.

## Abbreviations

The following abbreviations are used in this manuscript:

PEG	Polyethylene glycol
HDI	Hexamethylene diisocyanate
MD	Molecular dynamics
TPU	Thermoplastic polyurethane
Tg	Glass transition temperature
OPLS-AA	Optimized Potentials for Liquid Simulations—All Atom
OpenFF	Open Force Field
PolyParGen	Polymer Parameter Generator
GROMACS	GROningen MAchine for Chemical Simulations
PDB	Protein Data Bank
PME	Particle Mesh Ewald
LINCS	LINear Constraint Solver
PCA	Principal Component Analysis
ANOVA	analysis of variance
RANSAC	RANdom SAmple Consensus
HSD	Honest Significant Difference
FTIR	Fourier Transform Infrared
Rg	Radius of gyration
DSC	Differential scanning calorimetry

## References

[1] Das A., Mahanwar P. (2020). A Brief Discussion on Advances in Polyurethane Applications. Adv. Ind. Eng. Polym. Res..

[2] Shin E.J., Prasad C., Choi H.Y. (2025). Recent Advances in Thermoplastic Polyurethane-Based Composites, Properties, Synthesis and Its Applications. J. Ind. Eng. Chem..

[3] Yilgör I., Yilgör E., Wilkes G.L. (2015). Critical Parameters in Designing Segmented Polyurethanes and Their Effect on Morphology and Properties: A Comprehensive Review. Polymer.

[4] Zhao J., Zhu J., Zhang J., Huang Z., Qi D. (2024). Review of Research on Thermoplastic Self-Healing Polyurethanes. React. Funct. Polym..

[5] Devi R., Gupta P., Khatua C., Naskar K., Chattopadhyay S. (2025). Thermoplastic Polyurethane-Based Stimuli-Responsive Nanocomposites: A Review on Self-Healing and Shape Memory Properties. Polym. Eng. Sci..

[6] Randall D., Lee S. (2002). The Polyurethanes Book.

[7] Lunardon G., Sumida Y., Vogl O. (1980). Effects of Molecular Weight and Molecular Weight Distribution of Polyester Based Soft Segments on the Physical Properties of Linear Polyurethane Elastomers. Die Angew. Makromol. Chem..

[8] Klinedinst D.B., Yilgör I., Yilgör E., Zhang M., Wilkes G.L. (2012). The Effect of Varying Soft and Hard Segment Length on the Structure–Property Relationships of Segmented Polyurethanes Based on a Linear Symmetric Diisocyanate, 1,4-Butanediol and PTMO Soft Segments. Polymer.

[9] Ertem S.P., Yilgor E., Kosak C., Wilkes G.L., Zhang M., Yilgor I. (2012). Effect of Soft Segment Molecular Weight on Tensile Properties of Poly(Propylene Oxide) Based Polyurethaneureas. Polymer.

[10] Wang H., Li Y., Zhang H., Lang X., Wang X., Cao L., Zong C. (2025). Effects of Soft Segment Molecular Weight on the Properties of Recyclable Polyurethanes with Low-temperature Multiple Shape Memory. J. Polym. Sci..

[11] Koberstein J.T., Stein R.S. (1983). Small-angle X-ray Scattering Studies of Microdomain Structure in Segmented Polyurethane Elastomers. J. Polym. Sci. Polym. Phys. Ed..

[12] Garrett J.T., Siedlecki C.A., Runt J. (2001). Microdomain Morphology of Poly(Urethane Urea) Multiblock Copolymers. Macromolecules.

[13] Kojio K., Nozaki S., Takahara A., Yamasaki S. (2020). Influence of Chemical Structure of Hard Segments on Physical Properties of Polyurethane Elastomers: A Review. J. Polym. Res..

[14] Cheng B.-X., Gao W.-C., Ren X.-M., Ouyang X.-Y., Zhao Y., Zhao H., Wu W., Huang C.-X., Liu Y., Liu X.-Y. (2022). A Review of Microphase Separation of Polyurethane: Characterization and Applications. Polym. Test..

[15] Wilkes C.E., Yusek C.S. (1973). Investigation of Domain Structure in Urethan Elastomers by X-Ray and Thermal Methods. J. Macromol. Sci. Part B.

[16] Coleman M.M., Lee K.H., Skrovanek D.J., Painter P.C. (1986). Hydrogen Bonding in Polymers. 4. Infrared Temperature Studies of a Simple Polyurethane. Macromolecules.

[17] Yanagihara Y., Osaka N., Iimori S., Murayama S., Saito H. (2015). Relationship Between Modulus and Structure of Annealed Thermoplastic Polyurethane. Mater. Today Commun..

[18] Rahimzadeh R., Flanders M., Manas-Zloczower I. (2025). Optimization of the Annealing Procedure for Thermoplastic Polyurethane Systems Using in Situ Dynamic Mechanical Analysis. Phys. Fluids.

[19] Wang Z., Wang C., Zhao X., Yang X. (2025). Manipulating the Mechanical Properties of Thermoplastic Polyurethane via Regulating Hard Segment Aggregation. Macromolecules.

[20] Begenir A., Michielsen S., Pourdeyhimi B. (2009). Crystallization Behavior of Elastomeric Block Copolymers: Thermoplastic Polyurethane and Polyether-block-amide. J. Appl. Polym. Sci..

[21] Stribeck A., Eling B., Pöselt E., Malfois M., Schander E. (2019). Melting, Solidification, and Crystallization of a Thermoplastic Polyurethane as a Function of Hard Segment Content. Macromol. Chem. Phys..

[22] Nunes R.W., Martin J.R., Johnson J.F. (1982). Influence of Molecular Weight and Molecular Weight Distribution on Mechanical Properties of Polymers. Polym. Eng. Sci..

[23] Castagna A.M., Pangon A., Choi T., Dillon G.P., Runt J. (2012). The Role of Soft Segment Molecular Weight on Microphase Separation and Dynamics of Bulk Polymerized Polyureas. Macromolecules.

[24] Huang H., Pang H., Huang J., Yu P., Li J., Lu M., Liao B. (2021). Influence of Hard Segment Content and Soft Segment Length on the Microphase Structure and Mechanical Performance of Polyurethane-Based Polymer Concrete. Constr. Build. Mater..

[25] Oguz O., Koutsoumpis S.A., Simsek E., Yilgor E., Yilgor I., Pissis P., Menceloglu Y.Z. (2017). Effect of Soft Segment Molecular Weight on the Glass Transition, Crystallinity, Molecular Mobility and Segmental Dynamics of Poly(Ethylene Oxide) Based Poly(Urethane–Urea) Copolymers. RSC Adv..

[26] Saraf V.P., Glasser W.G., Wilkes G.L., McGrath J.E. (1985). Engineering Plastics from Lignin. VI. Structure–Property Relationships of PEG-containing Polyurethane Networks. J. Appl. Polym. Sci..

[27] Thring R.W., Ni P., Aharoni S.M. (2004). Molecular weight effects of the soft segment on the ultimate properties of lignin-derived polyurethanes. Int. J. Polym. Mater. Polym. Biomater..

[28] Hernandez R., Weksler J., Padsalgikar A., Choi T., Angelo E., Lin J.S., Xu L.-C., Siedlecki C.A., Runt J. (2008). A Comparison of Phase Organization of Model Segmented Polyurethanes with Different Intersegment Compatibilities. Macromolecules.

[29] Fragiadakis D., Runt J. (2013). Molecular Dynamics of Segmented Polyurethane Copolymers: Influence of Soft Segment Composition. Macromolecules.

[30] Jouibari I.S., Haddadi-Asl V., Mirhosseini M.M. (2019). A Novel Investigation on Micro-Phase Separation of Thermoplastic Polyurethanes: Simulation, Theoretical, and Experimental Approaches. Iran. Polym. J..

[31] Yildirim E., Yurtsever M., Wilkes G.L., Yilgör I. (2016). Effect of Intersegmental Interactions on the Morphology of Segmented Polyurethanes with Mixed Soft Segments: A Coarse-Grained Simulation Study. Polymer.

[32] Petrović Z.S., Budinski-Simendić J. (1985). Study of the Effect of Soft-Segment Length and Concentration on Properties of Polyetherurethanes. I. The Effect on Physical and Morphological Properties. Rubber Chem. Technol..

[33] Nakamae K., Nishino T., Asaoka S., Sudaryanto (1996). Microphase Separation and Surface Properties of Segmented Polyurethane—Effect of Hard Segment Content. Int. J. Adhes. Adhes..

[34] D’Arlas B.F., Rueda L., de la Caba K., Mondragon I., Eceiza A. (2008). Microdomain Composition and Properties Differences of Biodegradable Polyurethanes Based on MDI and HDI. Polym. Eng. Sci..

[35] Jin X., Guo N., You Z., Tan Y. (2020). Design and Performance of Polyurethane Elastomers Composed with Different Soft Segments. Materials.

[36] Liu B., Niu Z., Wang Z., Wang Y., Zou H., Zhao X., Hu S. (2025). Effect of Hard Segment Content on the Phase Separation and Properties of Hydroxyl-Terminated Polybutadiene Thermoplastic Polyurethane. Polym. Test..

[37] Yilgor I., Eynur T., Yilgor E., Wilkes G.L. (2009). Contribution of Soft Segment Entanglement on the Tensile Properties of Silicone–Urea Copolymers with Low Hard Segment Contents. Polymer.

[38] Didovets Y., Brela M.Z. (2025). Structure–Property Relationship between Hard Segments of Shape Memory Polyurethane Copolymers and Interchain Hydrogen Bonds: A Comprehensive Theoretical Study. J. Phys. Chem. B.

[39] Das S., Cox D.F., Wilkes G.L., Klinedinst D.B., Yilgor I., Yilgor E., Beyer F.L. (2007). Effect of Symmetry and H-bond Strength of Hard Segments on the Structure-Property Relationships of Segmented, Nonchain Extended Polyurethanes and Polyureas. J. Macromol. Sci. Part B.

[40] Yilgor I., Yilgor E., Guler I.G., Ward T.C., Wilkes G.L. (2006). FTIR Investigation of the Influence of Diisocyanate Symmetry on the Morphology Development in Model Segmented Polyurethanes. Polymer.

[41] Fernández-d’Arlas B., Ramos J.A., Saralegi A., Corcuera M., Mondragon I., Eceiza A. (2012). Molecular Engineering of Elastic and Strong Supertough Polyurethanes. Macromolecules.

[42] Bejagam K.K., Iverson C.N., Marrone B.L., Pilania G. (2020). Molecular Dynamics Simulations for Glass Transition Temperature Predictions of Polyhydroxyalkanoate Biopolymers. Phys. Chem. Chem. Phys..

[43] Jorgensen W.L., Ghahremanpour M.M., Saar A., Tirado-Rives J. (2024). OPLS/2020 Force Field for Unsaturated Hydrocarbons, Alcohols, and Ethers. J. Phys. Chem. B.

[44] Jorgensen W.L., Maxwell D.S., Tirado-Rives J. (1996). Development and Testing of the OPLS All-Atom Force Field on Conformational Energetics and Properties of Organic Liquids. J. Am. Chem. Soc..

[45] Mobley D.L., Bannan C.C., Rizzi A., Bayly C.I., Chodera J.D., Lim V.T., Lim N.M., Beauchamp K.A., Slochower D.R., Shirts M.R. (2018). Escaping Atom Types in Force Fields Using Direct Chemical Perception. J. Chem. Theory Comput..

[46] Wang J., Wolf R.M., Caldwell J.W., Kollman P.A., Case D.A. (2004). Development and Testing of a General Amber Force Field. J. Comput. Chem..

[47] Vanommeslaeghe K., MacKerell A.D. (2012). Automation of the CHARMM General Force Field (CGenFF) I: Bond Perception and Atom Typing. J. Chem. Inf. Model..

[48] Sun H. (1998). COMPASS: An Ab Initio Force-Field Optimized for Condensed-Phase Applications Overview with Details on Alkane and Benzene Compounds. J. Phys. Chem. B.

[49] Doseva V., Shenkov S., Baranovsky V.Y. (1997). Complex Formation Between Polymethacrylic Acid and Copolymers of Adipic Acid with Poly(Ethylene Glycol) in Aqueous Solution. Polymer.

[50] Yabe M., Mori K., Ueda K., Takeda M. (2019). Development of PolyParGen Software to Facilitate the Determination of Molecular Dynamics Simulation Parameters for Polymers. J. Comput. Chem. Jpn. Int. Ed..

[51] Horton J.T., Boothroyd S., Wagner J., Mitchell J.A., Gokey T., Dotson D.L., Behara P.K., Ramaswamy V.K., Mackey M., Chodera J.D. (2022). Open Force Field BespokeFit: Automating Bespoke Torsion Parametrization at Scale. J. Chem. Inf. Model..

[52] Smith D.G.A., Burns L.A., Simmonett A.C., Parrish R.M., Schieber M.C., Galvelis R., Kraus P., Kruse H., Remigio R.D., Alenaizan A. (2020). PSI4 1.4: Open-Source Software for High-Throughput Quantum Chemistry. J. Chem. Phys..

[53] Abraham M.J., Murtola T., Schulz R., Páall S., Smith J.C., Hess B., Lindah E. (2015). Gromacs: High Performance Molecular Simulations through Multi-Level Parallelism from Laptops to Supercomputers. Softwarex.

[54] Hess B., Bekker H., Berendsen H.J.C., Fraaije J.G.E.M. (1997). LINCS: A Linear Constraint Solver for Molecular Simulations. J. Comput. Chem..

[55] Darden T., York D., Pedersen L.G. (1993). Particle Mesh Ewald: An N⋅log(N) Method for Ewald Sums in Large Systems. J. Chem. Phys..

[56] Bussi G., Donadio D., Parrinello M. (2007). Canonical Sampling through Velocity Rescaling. J. Chem. Phys..

[57] Bernetti M., Bussi G. (2020). Pressure Control Using Stochastic Cell Rescaling. J. Chem. Phys..

[58] Gowers R., Linke M., Barnoud J., Reddy T., Melo M., Seyler S., Domański J., Dotson D., Buchoux S., Kenney I. MDAnalysis: A Python Package for the Rapid Analysis of Molecular Dynamics Simulations. Proceedings of the 15th Python in Science Conference.

[59] Michaud-Agrawal N., Denning E.J., Woolf T.B., Beckstein O. (2011). MDAnalysis: A Toolkit for the Analysis of Molecular Dynamics Simulations. J. Comput. Chem..

[60] Jolliffe I.T., Cadima J. (2016). Principal Component Analysis: A Review and Recent Developments. Philos. Trans. R. Soc. A Math. Phys. Eng. Sci..

[61] Patrone P.N., Dienstfrey A., Browning A.R., Tucker S., Christensen S. (2016). Uncertainty Quantification in Molecular Dynamics Studies of the Glass Transition Temperature. Polymer.

[62] Kemppainen J., Odegard G., Muzzy T., Wavrunek T. (2025). Understanding and Interpreting Stress-Strain Curves from Molecular Dynamics Simulation of Amorphous Polymers. ChemRxiv.

[63] Fischler M.A., Bolles R.C. (1987). Chapter 7—Matching, Model Fitting, Deduction, and Information Integration. Random Sample Consensus: A Paradigm for Model Fitting with Applications to Image Analysis and Automated Cartography. Readings in Computer Vision.

