# Supplementary material for: Benchmarking OPLS-AA and OpenFF for HDI–PEG Thermoplastic Polyurethanes with Varying Soft-Segment Length
Source: Molecules. 2026 Apr 10;31(8):1259. doi: 10.3390/molecules31081259 (PMC13118691; doi:10.3390/molecules31081259)
Supplement: Supplementary file 1 [file molecules-31-01259-s001.zip › molecules-4226108-supplementary.pdf]

Supplementary Materials

Benchmarking OPLS-AA and OpenFF for HDI–PEG Thermoplastic Polyurethanes with Varying Soft-Segment Length

Francesco Blasina [1], Tetiana Bubon [1,2], Francesco Cristiano [3], Giovanna G. Buonocore [3], Marino Lavorgna [3], Sabrina Pricl [1,4], Mariamelia Stanzione [3], Domenico Marson [1,*], and Erik Laurini [1]

[1] Molecular Biology and Nanotechnology Laboratory (MolBNL@UniTs), Department of Engineering and Architecture (DIA), University of Trieste, Piazzale Europa 1, 34127 Trieste, Italy.
[2] Bogolyubov Institute for Theoretical Physics of the National Academy of Sciences of Ukraine, 14-b Metrolohichna Str., Kyiv, 03143, Ukraine.
[3] Institute of Polymers, Composites and Biomaterials – CNR, P.le E. Fermi 1, 80055 Portici (Naples), Italy.
[4] Department of General Biophysics, Faculty of Biology and Environmental Protection, University of Lodz, ul. Pomorska 141/143, 90-236 Łódź, Poland.
* Correspondence: domenico.marson@dia.units.it; Tel.: +39 040 5583437

S1. Details on experimental characterization of HDI–PEG Film Samples 2
S1.1. FTIR Absorption Spectra of TPU Films 2
S1.2. Thermal degradation analysis 4
S2. MD Simulations details 6

S1. Details on experimental characterization of HDI–PEG Film Samples

S1.1. FTIR Absorption Spectra of TPU Films

The Fourier Transform Infrared (FTIR) spectra of synthesized films obtained from polyethylene glycol (PEG) (Mw = 400, 1000, and 1500 g/mol) and hexamethylene diisocyanate (HDI) confirmed the successful formation of urethane linkages and, consequently, the thermoplastic polyurethane (TPU) networks. All spectra exhibited a broad absorption band around 3300–3350 cm^{-1}, attributed to N–H stretching vibrations, and, thus, the related characteristic C=O stretching band of urethane groups at approximately 1700–1725 cm^{-1}. The presence of the amide II band at 1530–1535 cm^{-1}, arising from N–H bending coupled with C–N stretching, is indicative of hydrogen-bonded urethane groups. The position and relative intensity of this band suggest the establishment of intermolecular hydrogen bonding interactions, which are responsible for the formation of physically crosslinked hard-segment domains within the TPU matrix. Notably, variations in the relative intensity and slight shifts of the carbonyl (amide I, ~1700–1725 cm^{-1}) were observed as PEG molecular weight increased. These changes reflect differences in the extent of hydrogen bonding and microphase separation between hard and soft segments. In TPUs, increasing the molecular weight of the soft segment (PEG) generally promotes greater chain mobility and enhances phase segregation, leading to more defined hard-segment domains. The observed spectral modifications therefore suggest that PEG chain length influences the organization and packing of urethane hard domains within the polymer network. The complete disappearance of the isocyanate absorption band at ~2250 cm^{-1} confirms the complete consumption of the –N=C=O groups, indicating an efficient polymerization process. In addition, the bands at 2850–2950 cm^{-1} correspond to aliphatic –CH_2 stretching vibrations of the polymer backbone, while the strong absorptions at 1100–1150 cm^{-1} are attributed to C–O–C stretching of the PEG soft segments. A slight shift in both carbonyl and ether bands with increasing PEG molecular weight support changes in segmental mobility and intermolecular interactions. These results are consistent with the expected chemical structure and also provide evidence of molecular-level structural differences induced by varying PEG chain length. Figure S1.1 shows the FTIR spectra of the synthesized TPU films prepared using PEG with molecular weights of 400 g/mol, 1000 g/mol, and 1500 g/mol.

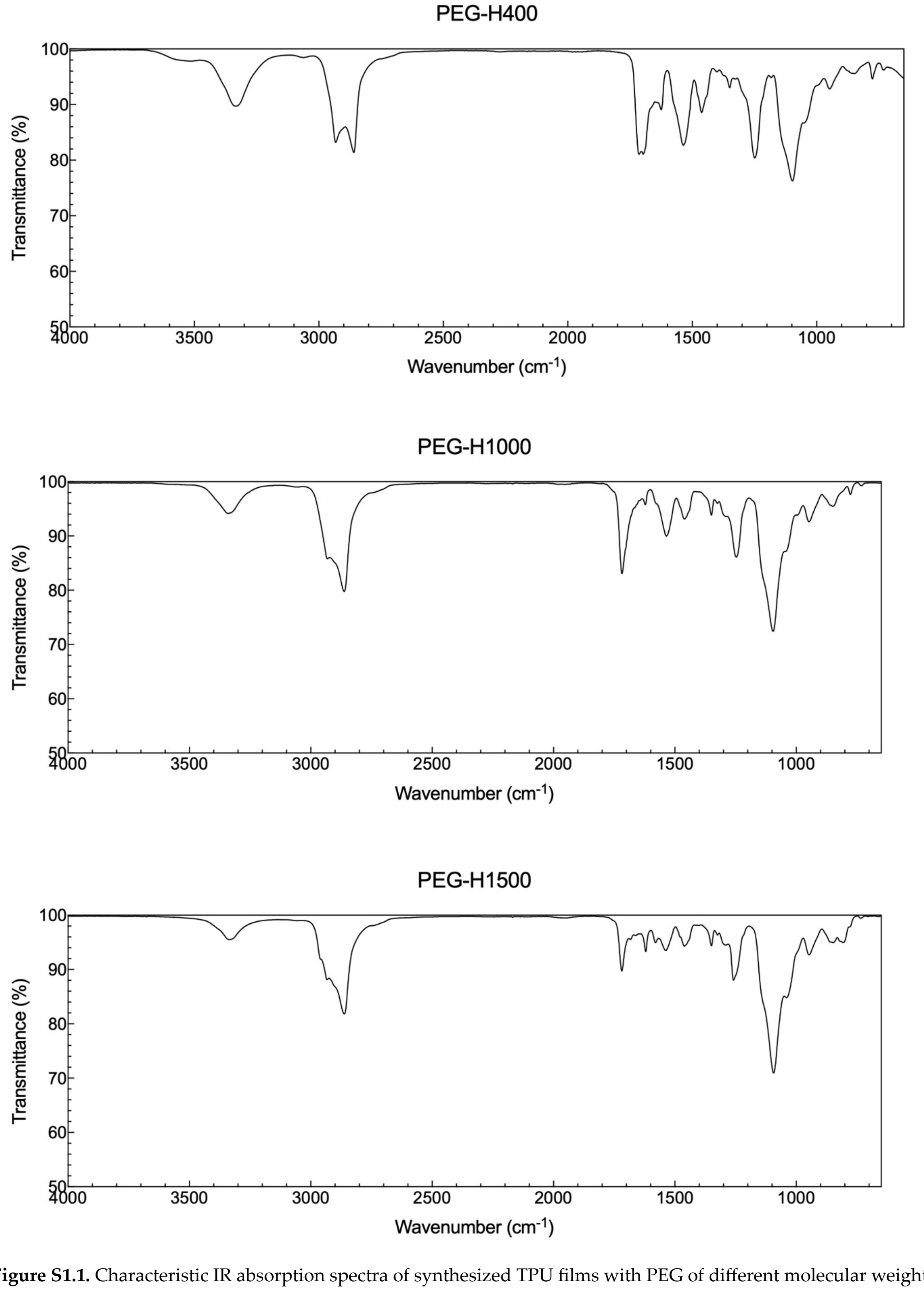

Figure S1.1. Characteristic IR absorption spectra of synthesized TPU films with PEG of different molecular weight.

S1.2. Thermal degradation analysis

Thermogravimetric (TGA) and derivative thermogravimetry (DTG) analysis were performed to evaluate the thermal stability and degradation behavior of PEG-based TPUs. All samples exhibited a characteristic two-step degradation profile, typical of poly(ether)-based TPUs, reflecting the sequential decomposition of urethane hard segments followed by degradation of the polyether soft segments.

The PEG-H400 sample (Figure S1.2, left panel) showed two DTG maxima of comparable intensity at approximatively 350 °C and 445 °C. The first degradation step is assigned to the urethane linkage dissociation and decomposition of hard-segment domains, involving carbamate cleavage and formation of isocyanate- and alcohol-derived fragments. The second peak corresponds to main chain scission of the PEG soft segments. The similar intensity of the two degradation events suggests a relatively balanced contribution of hard and soft domains to the overall mass loss, this behavior is consistent with a less pronounced phase separation and a comparatively higher relative hard-segment content, leading to a more heterogeneous microstructure with thermally stable domains persisting to higher temperatures.

In contrast, the PEG-H1000 sample (Figure S1.2, central panel) displayed two DTG maxima at ~300 °C and ~370 °C, with the second peak markedly more intense than the first. The lower-temperature event is assigned to urethane dissociation, while the dominant second peak corresponds to degradation of the polyether soft phase. The shift toward lower degradation temperatures compared to PEG-H400, together with the predominance of the second DTG maximum, indicates that soft-segment decomposition governs the overall thermal behavior. This trend is consistent with the increased soft-segment fraction and enhanced chain mobility associated with higher PEG molecular weight, which promote phase segregation and facilitate earlier degradation of the polyether domains once the hard segments have dissociated.

In the case of PEG-H1500 (Figure S1.2, right panel), two DTG maxima are shown at ~315 °C and ~385 °C, mirroring the trend observed for PEG1000: a minor urethane-related event followed by a significantly more intense soft-segment degradation peak. The slightly higher degradation temperatures relative to PEG-H1000 suggest marginally improved thermal resistance, which may be rationalized by differences in microphase organization and hydrogen-bonding density as the soft-segment length increases.

In conclusion, increasing PEG molecular weight progressively shifts the degradation behavior toward a soft-segment-dominated mechanism (second DTG peak much more

intense than the first one), while the peak temperatures fall within the expected ranges for urethane dissociation (~300–350 °C) and polyether backbone scission (~370–445 °C). The PEG-H400 system retains a pronounced high-temperature degradation event at 445 °C with intensity comparable to the first step, whereas PEG-H1000 and PEG-H1500 exhibit more unified soft-segment-driven decay profile, consistent with their higher relative polyether fraction.

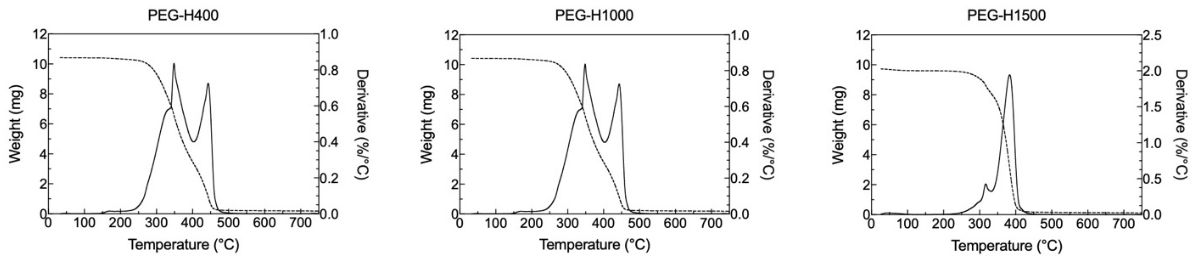

Figure S1.2. Thermogravimetric (TGA, dotted lines) and derivative thermogravimetry (DTG , full lines) curves of synthesized TPU films with PEG of different molecular weights.

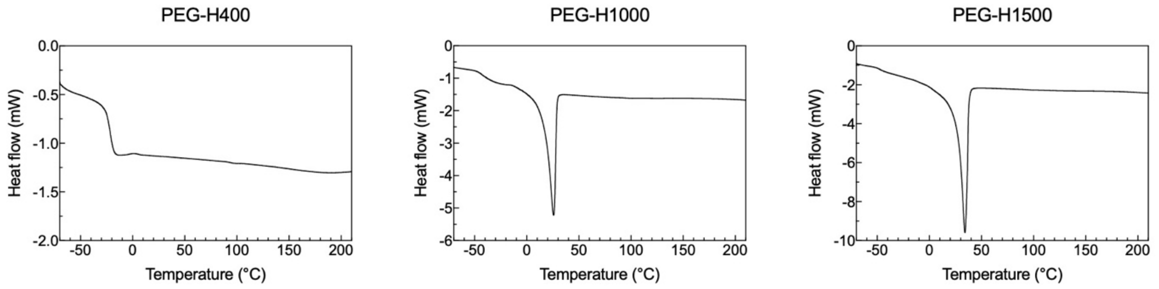

Figure S1.3. Differential scanning calorimetric (DSC) thermogram of synthesized TPU films with PEG of different molecular weights.

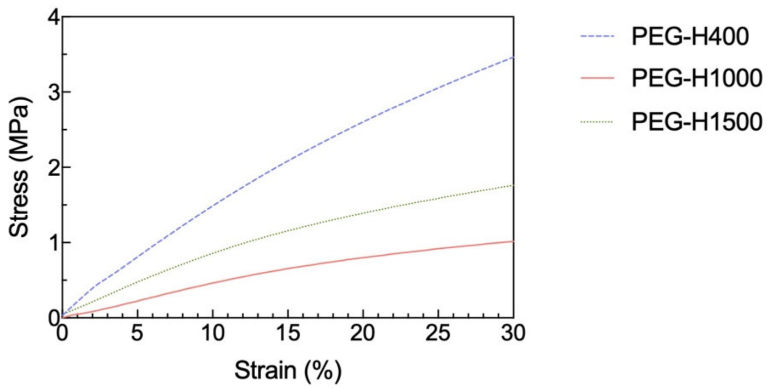

Figure S1.4. Stress-strain curve obtained from DMA mechanical analysis on the synthesized TPU films with PEG of different molecular weights.

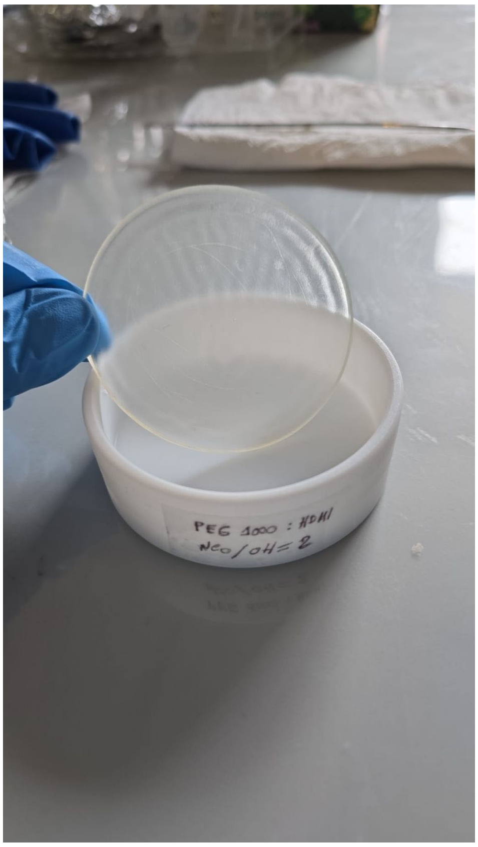

Figure S1.5. Representative photograph of the obtained polymer films suitable for thermo-mechanical characterization.

Table S1.1. Formulation of HDI–PEG polymer films.

Sample name	PEG (M_w)	PEG (g)	HDI (g)
PEG-H400	400	5	4.24
PEG-H1000	1000	5	1.68
PEG-H1500	1500	5	1.11

S2. MD Simulations details

Table S2.1. Details of simulated systems.

System name	Soft-segment M_W (g/mol)	# of chains	Starting box size (nm)
PEG-H400	400	130	4.83
PEG-H800	800	78	4.83
PEG-H1000	1000	64	4.83
PEG-H1500	1500	40	4.84
PEG-H2000	2000	30	4.81

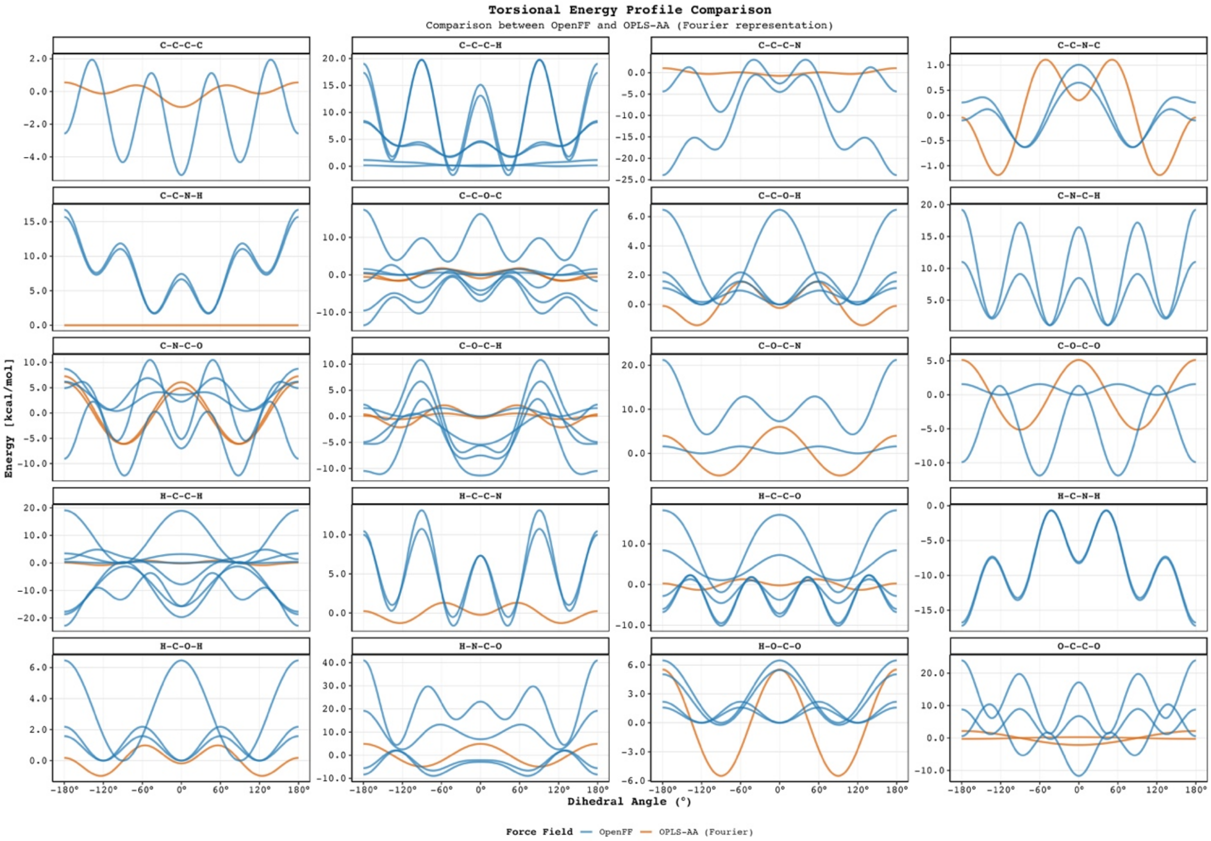

Figure S2.1. Energy profiles of torsion angles parameterized via the BespokeFit workflow in OpenFF, in comparison with the torsion profiles applied in OPLS-AA.

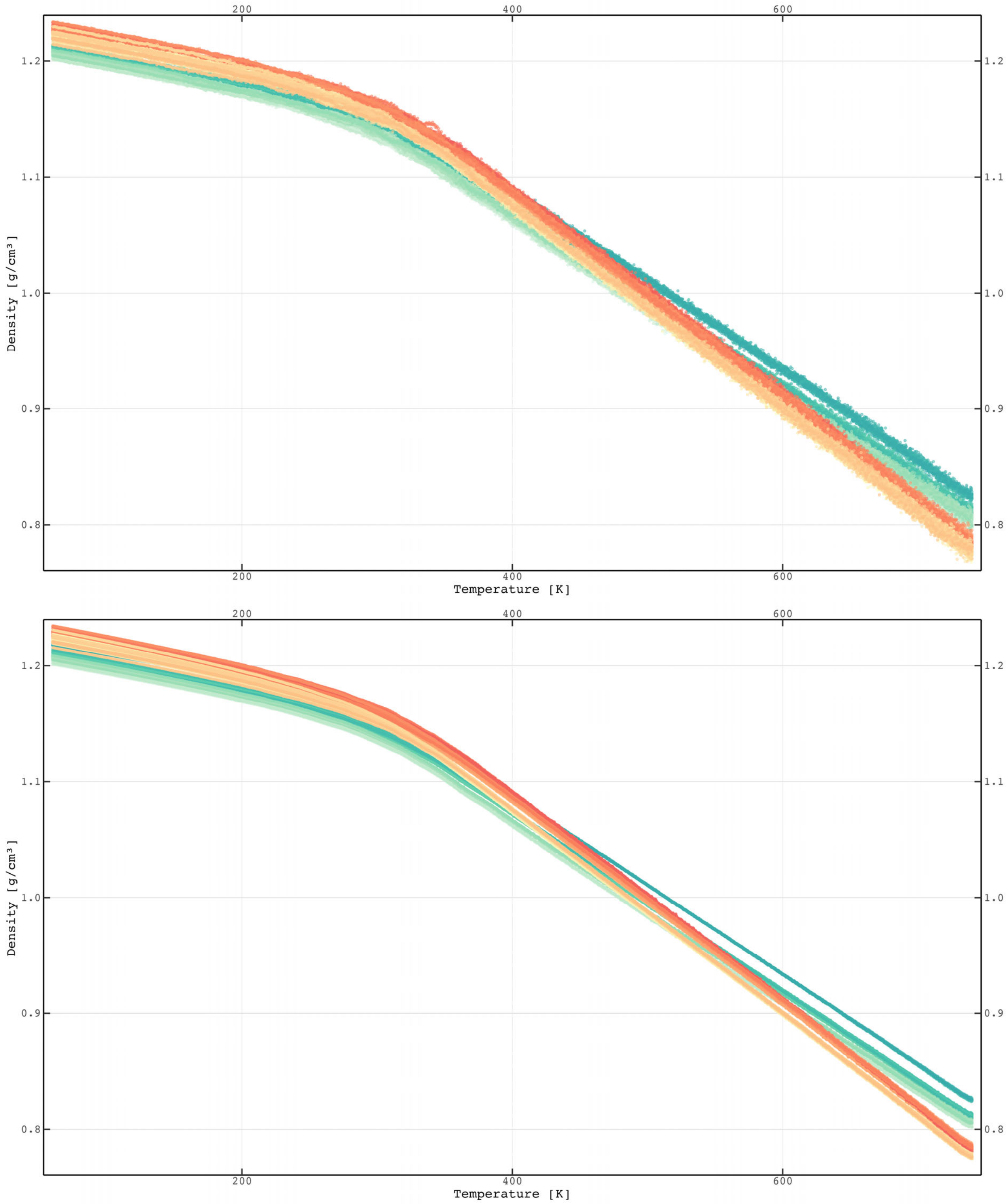

Figure S2.2. Density–temperature curves before (upper panel) and after (lower panel) PCA filtering, illustrating suppression of high-temperature fluctuations while preserving the underlying trend used to determine Tg. OPLS-AA and OpenFF results are shown in shades of orange and cyan, respectively.

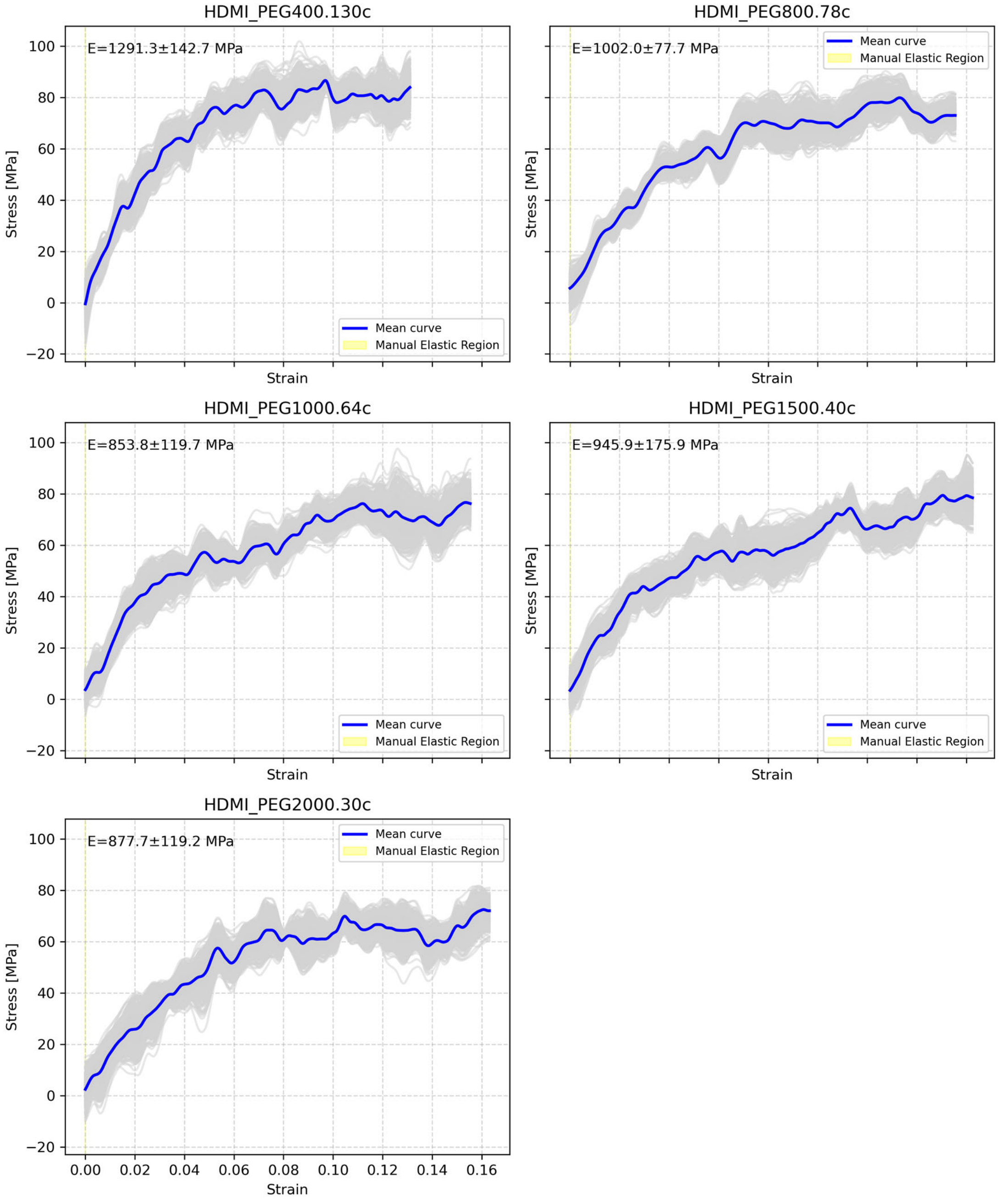

Figure S2.3. Stress–strain curves calculated from MD simulations using the OPLS-AA force field for polymer systems with different PEG molecular weights. The gray shaded regions represent bootstrap-resampled stress–strain curves, while the blue lines denote the corresponding bootstrap-averaged curves.

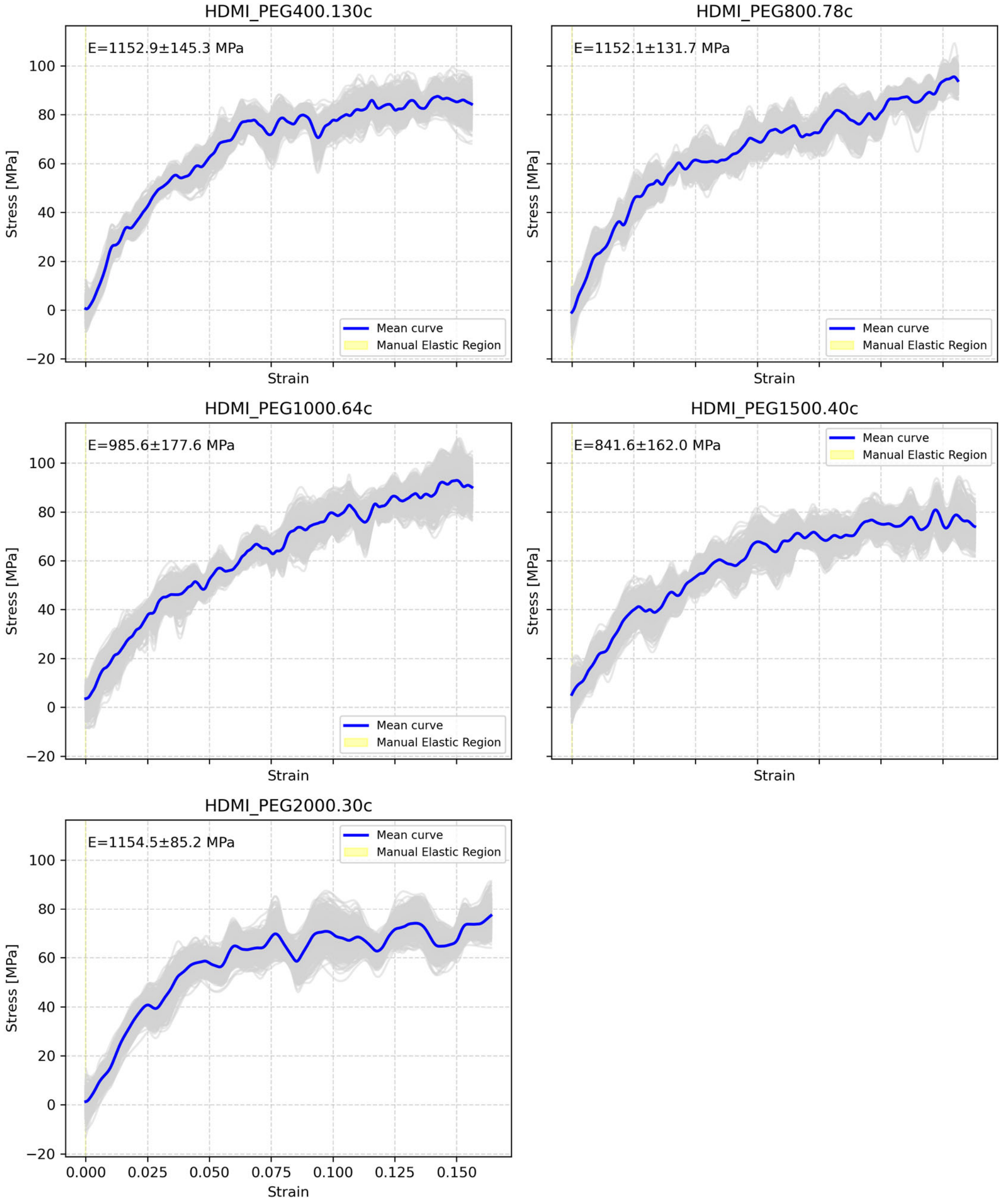

Figure S2.4. Stress–strain curves calculated from MD simulations using the OpenFF force field for polymer systems with different PEG molecular weights. The gray shaded regions represent bootstrap-resampled stress–strain curves, while the blue lines denote the corresponding bootstrap-averaged curves.

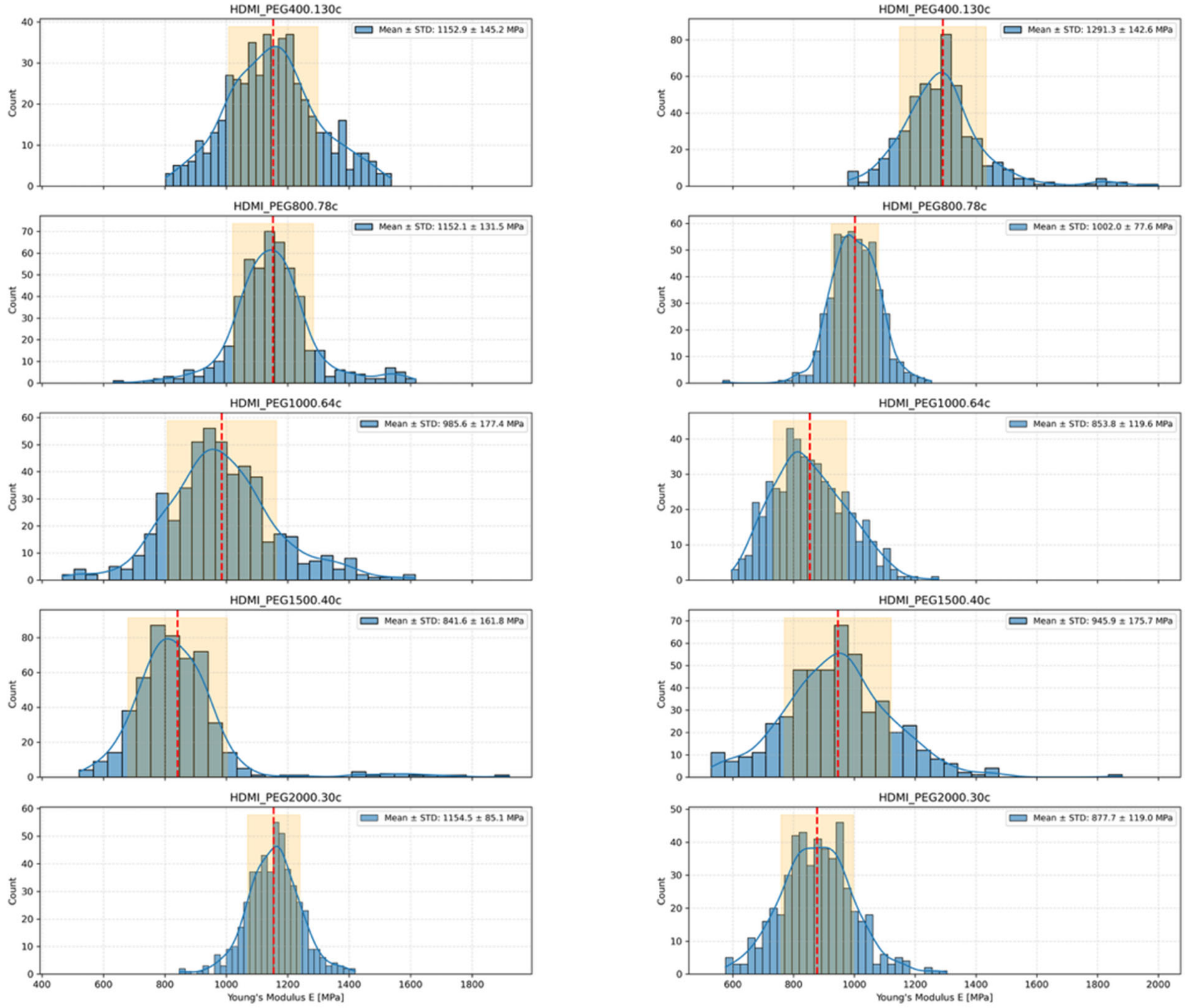

Figure S2.5. Distributions of the elastic modulus values obtained from bootstrap resampling of MD simulation data using the OpenFF (left panels) and OPLS-AA (right panels) force fields for polymer systems with different PEG molecular weights.
